# Hormonally-regulated proteins in breast secretions are markers of target organ sensitivity

**DOI:** 10.1054/bjoc.1999.0926

**Published:** 2000-01-18

**Authors:** C Harding, O Osundeko, L Tetlow, E B Faragher, A Howell, N J Bundred

**Affiliations:** Departments of Surgery, Chemical Pathology and Statistics, University Hospital of South Manchester, Nell Lane, West Didsbury, Manchester, M20 8LR, UK; CRC Medical Oncology, Christie Hospital NHS Trust, Manchester, UK

**Keywords:** breast

## Abstract

Anti-oestrogen therapy is being used in an attempt to prevent breast cancer but no intermediate end points of the effect of tamoxifen on the normal breast are available. Therefore, the purpose of this study was to develop a physiological measure of oestrogen action on the breast. We measured oestrogen-stimulated and -inhibited proteins in breast secretions from women on and off anti-oestrogen therapy. Two oestrogen-stimulated proteins (pS2 and cathepsin D) and oestrogen-inhibited proteins (CP15, gross cystic disease fluid protein 15; Apo ,: apolipoprotein D) were measured. Premenopausal women had significantly higher pS2 and cathepsin D in association with lower Apo D and CP15 secretion levels compared to post-menopausal women. Sequential nipple aspirates from women treated with the luteinizing hormone releasing hormone agonist goserelin (*n* = 9), tamoxifen (*n* = 9) and hormone replacement therapy (HRT) (*n* = 26) were measured. Following treatment with goserelin, median nipple secretion levels of pS2 fell (*P*< 0.02) and Apo D and CP15 rose significantly (*P*< 0.03 and *P*< 0.05 respectively). Similar changes were seen on tamoxifen therapy but not in untreated control women. Treatment with HRT resulted in a rise of pS2 (*P*< 0.001) and a fall in Apo D (*P*< 0.05). Measurement of pS2 and Apo D in nipple aspirates may prove useful intermediate end point of breast responsiveness to anti-oestrogens. © 2000 Cancer Research Campaign


*British Journal of Cancer* (2000) **82**(2), 354–360
© 2000 Cancer Research Campaign
Article no. bjoc.1999.0926

### Hormonally-regulated proteins in breast secretions are markers of target organ sensitivity

**C Harding[1], O Osundeko[2], L Tetlow[2], EB Faragher[3], A Howell[4] and NJ Bundred[1]**

Departments of [1]Surgery, [2]Chemical Pathology and [3]Statistics, University Hospital of South Manchester, Nell Lane, West Didsbury, Manchester M20 8LR, UK; [4]CRC Medical Oncology, Christie Hospital NHS Trust, Manchester, UK

**Summary** Anti-oestrogen therapy is being used in an attempt to prevent breast cancer but no intermediate end points of the effect of tamoxifen on the normal breast are available. Therefore, the purpose of this study was to develop a physiological measure of oestrogen action on the breast. We measured oestrogen-stimulated and -inhibited proteins in breast secretions from women on and off anti-oestrogen therapy. Two oestrogen-stimulated proteins (pS2 and cathepsin D) and oestrogen-inhibited proteins (CP15, gross cystic disease fluid protein 15; Apo ,: apolipoprotein D) were measured. Premenopausal women had significantly higher pS2 and cathepsin D in association with lower Apo D and CP15 secretion levels compared to post-menopausal women. Sequential nipple aspirates from women treated with the luteinizing hormone releasing hormone agonist goserelin ($n$ = 9), tamoxifen ($n$ = 9) and hormone replacement therapy (HRT) ($n$ = 26) were measured. Following treatment with goserelin, median nipple secretion levels of pS2 fell ($P < 0.02$) and Apo D and CP15 rose significantly ($P < 0.03$ and $P < 0.05$ respectively). Similar changes were seen on tamoxifen therapy but not in untreated control women. Treatment with HRT resulted in a rise of pS2 ($P < 0.001$) and a fall in Apo D ($P < 0.05$). Measurement of pS2 and Apo D in nipple aspirates may prove useful intermediate end point of breast responsiveness to anti-oestrogens. © 2000 Cancer Research Campaign

**Keywords:** breast

Oestrogen is the major steroid hormone which promotes normal breast epithelial cell growth and is believed to be important in the development of human breast cancer (Key and Pike, 1988). Early oophorectomy reduces the risk of breast cancer (Bullbrook et al, 1986; MacMahon et al, 1986; Key and Pike, 1988) and the administration of exogenous oestrogens increases the risk of the disease (Bergkvist et al, 1989; Hunt et al, 1987). Despite extensive study, however, little conclusive evidence has been published that serum oestrogen levels predict an individual's breast cancer risk. Plasma levels of oestrogen fall after the menopause whilst breast cancer risk rises in the same age group (Key and Pike, 1988). Other hormones may also influence the breast epithelium since low urinary and serum androgens are associated with increased breast cancer incidence (Zumoff, 1988), whilst hypophysectomy prevents the carcinogenic effects of oestrogen on the murine female breast (Lippsett and Bergenske, 1960).

Since serum levels of oestrogen in women at risk or with breast cancer are similar to age-matched controls (MacMahon et al, 1986; Key and Pike, 1988; Zumoff, 1988), it is possible that increase in the sensitivity of the breast epithelium contributes to the risk of breast cancer. Certain breasts may be inherently more sensitive to oestrogens than others.

Both oestrogen and androgens are concentrated by the breast (Ernster et al, 1987; Petrakis et al, 1987). In the post-menopausal breast tissue oestrogen levels are 40 times plasma levels and are equivalent to levels found in the premenopausal breast (Ernster et al, 1987; Petrakis et al, 1987).

*Received 5 January 1999*
*Revised 23 August 1999*
*Accepted 26 August 1999*

*Correspondence to:* NJ Bundred

Nipple aspirates (breast secretions) can be obtained by gentle breast compression or by applying negative pressure to the nipple using a Sartorius cup (Petrakis et al, 1981, 1987; Ernster et al, 1987). This nipple fluid represents secretion pooled in the lactiferous sinuses and proximal ducts. Such nipple aspirates (NA) can be produced from 70% of premenopausal and 40% of post-menopausal Caucasian women in our own and others' experience (Petrakis et al, 1981, 1987; Ernster et al, 1987; Wrensch et al, 1990).

A number of authors (Petrakis, 1986; Sanchez et al, 1992; Petrakis et al, 1993) have investigated breast duct epithelial secretions to determine the value of measurement of the chemical components in explaining the pathophysiology of breast conditions.

The breast epithelium responds to hormonal stimulation by producing a series of specific proteins and inhibiting production of others. The addition of oestrogen to the medium of MCF7 breast cancer cell lines has demonstrated that examples of oestrogen-induced proteins are pS2 and cathepsin D (Brown et al, 1984; Cavailles et al, 1988, 1989; Weaver et al 1988), whereas cyst protein 15 (CP15) and apolipoprotein D (Apo D) are oestrogen-inhibited but androgen-stimulated (Chalbos et al, 1987; Haagensen et al, 1990; Simard et al, 1990). Oestrogens markedly reduce Apo D secretion by ZR-75-1 and MCF-7 breast cancer cell lines: an effect reversed by androgens and anti-oestrogens (Chalbos et al, 1987; Simard et al, 1990).

Simard et al have shown that levels of both CP15 and Apo D secreted into the medium of cancer cell lines relate closely to the proliferative activity of the cell line, levels increasing when the cells are quiescent (Chalbos et al, 1987; Haagensen et al, 1990; Simard et al, 1990).

To attempt to produce a physiological measure of hormonal stimulation of the female breast (i.e. target organ sensitivity) we have measured all four proteins in NA from women with normal breasts, women with benign disease and women with breast pain treated with goserelin and tamoxifen. To confirm the oestrogen-responsiveness of the proteins measured we have also assessed the effect of hormone replacement therapy (HRT) on the concentrations of the four proteins in sequential nipple secretions. We show that, in general, oestrogen-stimulation is associated with an increase in pS2 and a decrease in Apo D and CP15.

### MATERIALS AND METHODS

### Patients

Women with no breast disease (*n* = 63; age range 21–68 years), benign breast disease (*n* = 53; age range 20–61 years) and breast pain (*n* = 18; age range 24–39 years) who attended symptomatic breast clinics at the University Hospital of South Manchester were studied. In the Palatine Centre, Manchester 26 women (age range 40–69 years), who were about to commence HRT, were recruited from the Menopause Clinic. All 26 women about to start HRT (combined HRT, *n* = 16; oestrogen only, *n* = 10) were self-referrals and had no intercurrent breast disease. All oral HRT preparations provided between 0.625 mg and 2.0 mg oestradiol daily or 50 μg oestradiol topically. All women gave informed consent. The study was approved by the Ethics Committee of the University Hospital of South Manchester and detailed information of reproductive status, history of breast disease, menopausal status, lactation and last menstrual period was provided by all women.

All premenopausal women had regular menstrual cycles lasting between 24–33 days and none (apart from the HRT group) were on medication likely to alter pituitary or ovarian function from baseline. There were no significant differences in mean age or reproductive history between the women with or without benign breast disease.

Benign breast disease was diagnosed from radiographic, echographic, cytological and histological studies. Among women with benign disease, 16 were post-menopausal, of whom eight had duct ectasia, three had a fibroadenoma, and miscellaneous cases (e.g. fibroadenosis), 36 were premenopausal, of whom 18 had breast pain, 11 had duct ectasia, four had breast cysts and three had fibrocystic masses. Women with spontaneous nipple discharge were excluded from the study. Women with mastalgia completed breast pain charts confirming cyclical mastalgia (defined as severe pain, unresponsive to evening primrose oil, for a minimum of 7 days per cycle) for a minimum of 4 months. Women meeting these criteria were offered treatment with goserelin or tamoxifen as appropriate.

There were 35 premenopausal and 28 post-menopausal women identified from the same clinics who had no evidence of breast disease. They presented with symptoms of breast lumps, nipple changes or concerns regarding potential risk of breast cancer (e.g. family history), but were found, after clinical assessment and radiological investigations, to have no abnormality and no increased breast cancer risk.

### Breast fluid collection

Nipple secretion specimens were obtained from each breast by bimanual, four quadrant compression, followed by Sartorius cup suction (Petrakis et al, 1981) if compression failed. Attempts were made to obtain specimens from both breasts. Secretion specimens were collected before any diagnostic study or surgical procedure was carried out on the breast. The fluid was collected into capillary tubes. The volume of neat nipple secretion was calculated by multiplying the length (in mm) of nipple fluid in the tube by the cross-sectional area of the lumen of the capillary tube. Volumes obtained varied from 0.67 to 106 μl. The capillary tubes were transferred into 1.5 ml test tubes and centrifuged at 6500 rpm for 5 min. Phosphate-buffered saline (PBS) solution was then added to the nipple secretion to a known dilution (between 1:25 and 1:300). The diluted specimen was then frozen at –20°C for later analysis.

### Assays

Apolipoprotein D (Apo D) was measured using a competitive polyclonal radioimmunoassay previously described by Haagensen and using the principles of Yalow and Berson (Sanchez et al, 1992). Fifty microlitres of standard Apo D or specimens were incubated with 200 μl of $^{125}$I-Apo D and 200 μl of rabbit anti-human Apo D serum (1:5000) for 4 h at 4°C. Apo D–antibody complexes were then precipitated by incubating the reaction mixture with 50 μl of donkey anti-rabbit coated microcrystalline cellulose (sac-cel) for 30 min at room temperature. Three millilitres of wash solution (0.9% sodium chloride and 0.05% Tween-20) were then added and the reaction mixture centrifuged at 2500 rpm for 20 min at 10°C. The supernatant was then discarded and bound $^{125}$I-Apo D–antibody complex was then counted using a gamma scintillation counter. The concentrations of Apo D in the specimens were inversely proportional to the radioactive counts obtained for the specimens. These concentrations were calculated from a standard curve plotted as % $B/B_o$ versus $\log_{10}$ standard concentration, where B = bound counts and $B_o$ = maximum counts bound. The standard curve was constructed using standard solutions of 10 000, 5000, 1000, 500 and 250 ng $ml^{-1}$ of Apo D. The sensitivity of the assay was 80 ng $ml^{-1}$. Intra-assay variation was less than 9% over the full working range and inter-assay variation was 12.4%, 8.2% and 9.7% at levels of 620 ng $ml^{-1}$, 1250 ng $ml^{-1}$ and 3030 ng $ml^{-1}$ respectively. Breast samples were analysed in duplicate (at two dilutions, 1:10 000 and 1:50 000) and the average concentration for each specimen was calculated. The Apo D concentration in the original, undiluted sample was then obtained and expressed in g $l^{-1}$. Purified apolipoprotein D and rabbit anti-apolipoprotein D were a kind gift from Dr DA Haagensen, Sacramento, USA.

Gross cystic disease fluid protein (CP15) was quantified using a novel competitive enzyme immunoassay in which purified CP15 (coated on to microtitre wells) and CP15 in standard or patient sample compete for binding to rabbit anti-CP15. A 96-well plate was coated with 100 μl of 250 ng $ml^{-1}$ CP15 in azide PBS solution and incubated at 4°C overnight. The coating was then discarded and each well was then washed with 200 μl of wash buffer twice and the buffer discarded. One hundred microlitres of bovine-specific antigen (BSA) in PBS was added to each well and the plate was again incubated overnight at 4°C and the BSA was discarded the next day. Standards of 125, 62, 31, 15, 7, 4 and 2 ng $ml^{-1}$ were used to construct the standard curve. Fifty microlitres of standards and specimens were then transferred to the plate wells in triplicate, and after adding 50 μl of 1:10 000 anti-CP15 to each well (except those wells allocated to test non-specific binding which were filled with 100 μl dilution buffer only), the plates were then incubated for a further 4 h at 4°C. The plate contents were again then discarded and 100 µl of 1:3000 dilution of anti-rabbit peroxidase-labelled IgG conjugate was added to each well and the plate was again incubated overnight at 4°C. After washing with wash buffer and citrate buffer and the contents discarded, 100 µl of OPD solution was then added to each plate and the plate stored in the dark for 5 min at room temperature. The colour reaction was then stopped with 100 µl 1M hydrochloric acid. The plate was read using a 492 nm filter on a plate reader. The standard curve was plotted as log [CP15] ng $ml^{-1}$ against mean absorbance and the CP15 concentrations for the specimens were then calculated and expressed in ng $ml^{-1}$.

Purified CP15 and rabbit anti-CP15 were a kind gift from Dr DA Haagensen, Sacramento, USA. The interassay variation was 15%.

pS2 was measured using a commercially available radioimmunoassay kit (CIS UK Ltd). Each specimen, standard and control was analysed in duplicate and results expressed in pmol $ml^{-1}$. The interassay variation was 7.27% and the intraassay variation was 3.03%.

Cathepsin D (cat D) was also measured using a commercially available radioimmunoassay kit (CIS UK Ltd). Each specimen, standard and control was analysed in duplicate and results expressed in fmol $ml^{-1}$. The interassay variation was 3.64% and the intra-assay variation was 5.67%.

Total protein was measured by a modification of the Bradford method. Standards were first prepared using BSA in phosphate buffered saline PBS at dilutions of 0.002–0.02 µg $ml^{-1}$. Samples were prepared by making 1:100 dilutions of the original diluted samples using PBS, giving assay samples at dilutions of 1:2500–1:30 000. Using a 96-well, flat-bottomed plate, 75 µl of sample or standard was mixed with 75 µl dye reagent concentrate (Bio-Rad, Munich) diluted to 1:5 with distilled water. After 5 min the protein concentration was calculated from a standard curve by reading the OD for each sample using a 595 nm filter on a plate reader. Total protein was calculated in g $l^{-1}$. The interassay variation was 8.2% and the intraassay variation was 6.2%.

### Statistical analysis

Non-parametric statistical analysis of between-group (benign versus normal) differences was assessed using Mann–Whitney *U*-test and Kruskal–Wallis tests. Sequential changes in secretion protein levels were compared using the Wilcoxon test. Data are presented as median ± interquartile range. Parametric data was analysed using the paired *t*-test.

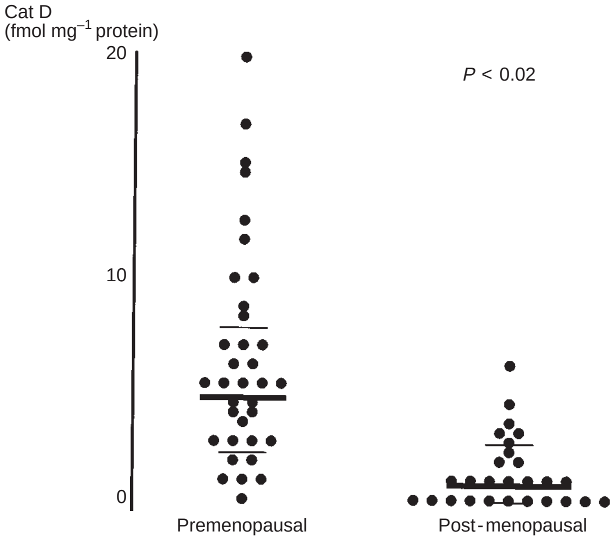

**Figure 1** Levels of cathepsin D in nipple aspirates from normal premenopausal women ($n$ = 35) were significantly higher than nipple aspirates from post-menopausal women ($n$ = 28, $P \leq 0.02$; Mann–Whitney *U*-test). Data are shown with thick horizontal line representing median value and thinner horizontal lines the interquartile ranges

### RESULTS

### Effect of the menopause on secretion of proteins

Cat D was higher in NA from normal premenopausal women compared to post-menopausal women ($P < 0.02$) (Figure 1 and Tables 1 and 2) There was no difference between NA pS2 concentrations from premenopausal compared to post-menopausal women with normal breasts (Tables 1 and 2). Apo D and CP15 were lower in breast secretions from premenopausal women compared to the post-menopausal group (Tables 1 and 2 and

**Table 1** Nipple secretion concentrations in premenopausal women

| | Normal | Benign | Cyclical mastalgia | | |
|---|---|---|---|---|---|
| | | | Before treatment | After goserelin | After tamoxifen |
| Number | 35 | 36 | 18 | 9 | 9 |
| Median [pS2] | 8.5 | 15.2 | 17.2 | 6.53 | 4.25 |
| Range (ng $mg^{-1}$ protein) | (1.4–107.4) | (0.2–104.9) | (0.9–155.9) | (0.12–38.7) | (0–25.03) |
| Median [Cat D] | 4.7 | 4.1 | 5.9 | 3.2 | 3.5 |
| Range (fmol $mg^{-1}$ protein) | (0.6–19.1) | (0–27.9) | (1.7–91.1) | (1.0–22.6) | (0.1–35.6) |
| Median [Apo D] | 159 | 178.2 | 47.15 | 525 | 303.5 |
| Range (µg $mg^{-1}$ protein) | (12–1833) | (8.9–791.6) | (8.9–339.9) | (34.4–861.3) | (76.5–485.2) |
| Median [CP15] | 33.05 | 25.44 | 29.1 | 99.3 | not done |
| Range (µg $mg^{-1}$ protein) | (0–188.9) | (0–183.4) | (0.8–169) | (35.5–146.1) | |

Nipple aspirate pS2 concentrations were significantly higher and Apo D concentrations were significantly lower in women with cyclical mastalgia compared with women with normal breasts ($P < 0.05$). Treatment with goserelin and tamoxifen significantly suppressed concentrations of pS2 ($P < 0.02$) and increased Apo D and CP15 ($P < 0.03$ and $P < 0.05$ respectively) in breast secretions. No significant effect was seen on cat D.

*© 2000 Cancer Research Campaign*

**Table 2** Nipple secretion concentrations in post-menopausal women before and after treatment with HRT

| | **Normal** | **Benign** | **Hormone replacement therapy** | |
|---|---|---|---|---|
| | | | **Before** | **After 6 months** |
| Number | 28 | 16 | 26 | 25 |
| Median [pS2] | 9.1 | 2.9 | 9.6 | 35.8 |
| Range (ng $mg^{-1}$ protein) | (1.4–148.9) | (0.1–7.7) | (0–130.71) | (10.4–130) |
| Median [Cat D] | 0.1 | 0.1 | 0.1 | 1.11 |
| Range (fmol $mg^{-1}$ protein) | (0–5.8) | (0.2–9.3) | (0.1–6.1) | (0.1–61) |
| Median [Apo D] | 257 | 140.5 | 193.5 | 167.5 |
| Range (μg $mg^{-1}$ protein) | (59–4314) | (27.2–1670.7) | (10.5–612.0) | (0–442.5) |
| Median [CP15] | 53.5 | 5.61 | 50.6 | 71.0 |
| Range (μg $mg^{-1}$ protein) | (0.2–159.1) | (0–68.86) | (0.004–159.1) | (0–212.7) |

Proteins in nipple secretions did not differ between normal breasts and cases of benign breast disease. HRT significantly increased pS2 ($P < 0.001$) and reduced Apo D ($P < 0.05$). Apo D and CP15 were significantly lower in benign breast disease compared with the normal breast ($P < 0.007$ and $P < 0.05$ respectively).

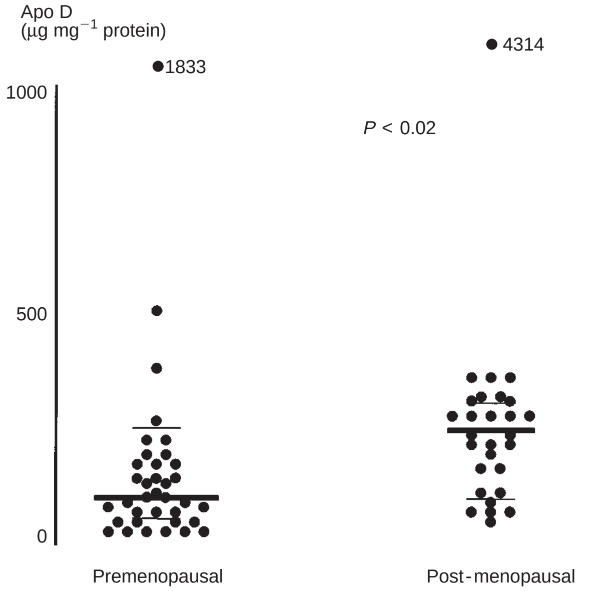

**Figure 2** Levels of apolipoprotein D in nipple aspirates from normal post-menopausal women with no breast disease ($n = 28$) were significantly higher than levels from normal premenopausal women ($n = 35$, $P \leq 0.02$)

Figure 2).

Analysis of NA from normal women revealed pS2 and Apo D levels inversely correlated ($P \leq 0.05$; Spearman's correlation coefficient). Amongst women with benign breast disease, pS2 concentrations in NA were higher in premenopausal (median 15.2 ng $mg^{-1}$ protein, range 0.2–104.9 ng $mg^{-1}$ protein) compared to post-menopausal secretions (median 2.9 ng $mg^{-1}$ protein, range 0.1–7.7, $P < 0.002$), but NA levels of cat D, Apo D and CP15 did not differ between premenopausal and post-menopausal women with benign disease (Tables 1 and 2).

In 35 women who provided NA bilaterally there was a good correlation of both Apo D and pS2 between breasts ($r = 0.9$; $P \leq 0.001$ for both). Additionally, 18 women gave sequential nipple aspirates at least 1 month apart whilst not on any therapy and levels did not change significantly between the two time points ($r = 0.99$ pS2, $r = 0.94$ Apo D). Geometric transformation of data revealed that levels in control women could vary as much as 31% (relative risk (RR) 1.03; 95% confidence interval (CI) 0.81–1.31) for pS2 and 71% (RR 1.06; 95% CI 0.66–1.71) for Apo D between women.

### Comparison of normal and benign breast disorders

In the premenopausal group, there were no significant differences in NA protein concentrations between normal women and those with benign breast disease (Table 1). Post-menopausal women with benign breast disease had lower Apo and concentrations compared to normal women ($P < 0.007$ and $P < 0.05$ respectively; see Tables 1 and 2). pS2 and cat D did not differ between the groups in post-menopausal women.

In patients with cyclical mastalgia, NA pS2 concentrations were significantly higher than in normal premenopausal women (Table 1, $P < 0.05$). Apo D concentrations were significantly lower in women with cyclical mastalgia compared with normal women (Table 1, $P < 0.04$).

### Cyclical mastalgia: effect of treatment with goserelin and tamoxifen

Treatment of patients with cyclical mastalgia with either goserelin or tamoxifen for 3 months significantly reduced pS2 to a median of 6.5 ng $mg^{-1}$ protein and 4.3 ng $mg^{-1}$ protein respectively ($P < 0.02$ and $P < 0.05$, Figure 3A). These treatments increased NA Apo D concentrations to 525 μg $mg^{-1}$ protein and 303.5 μg $mg^{-1}$ protein respectively ($P < 0.03$ and $P < 0.05$, Figure 3B and Table 1). The 300% decline in pS2 and the tenfold increase in Apo D detected with goserelin treatment was highly correlated ($r = -0.934$, $P < 0.001$) and contrasted with the small changes in the levels of the same proteins in sequential samples for control women. The ratio of pS2 to Apo D was significantly reduced from a median of 525 ng $\mu g^{-1}$ (range 50–2500 ng $\mu g^{-1}$) to 50 ng $\mu g^{-1}$ (0–410 ng $\mu g^{-1}$, $P < 0.008$). NA concentrations of CP15 also significantly increased

© 2000 Cancer Research Campaign

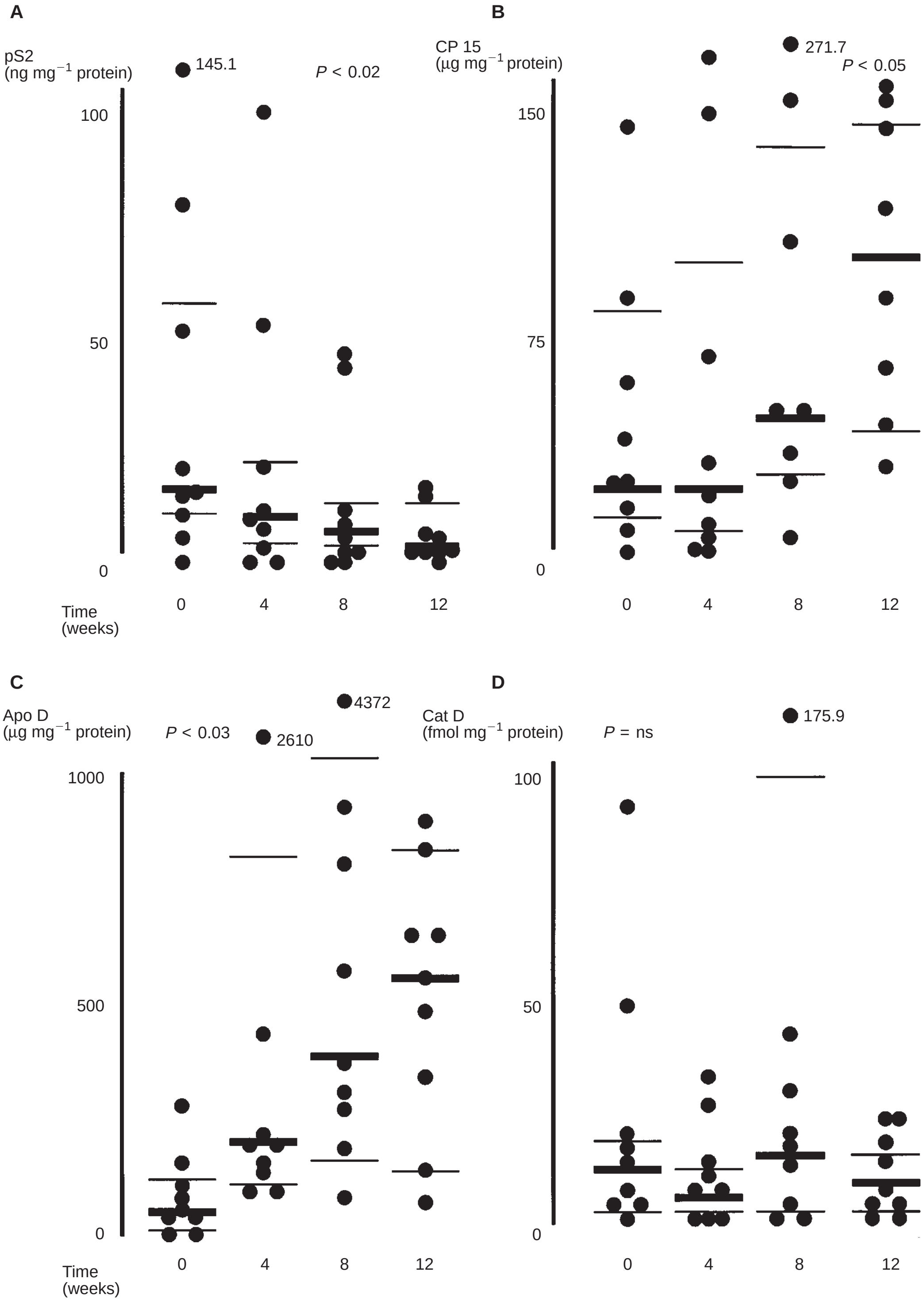

**Figure 3** The effect of goserelin (an LHRH agonist) treatment on nipple secretion levels of (**A**) pS2, **(B)** Apo D, **(C)** CP15 (otherwise known as GCDFP15) and **(D)** Cat D levels. Nine premenopausal women with breast pain underwent treatment with goserelin for 3 months. Prior to therapy nipple aspirate levels of pS2 were significantly higher and Apo D levels significantly lower than levels in aspirates from normal premenopausal breasts (see Table 1). On treatment a significant sequential fall in pS2 aspirate levels was mirrored by a significant rise in Apo D and CP15 levels. The correlation between pS2 and Apo D aspirate levels in the individual women was highly significant ($r = -0.93$; $P \leq 0.001$)

after treatment with goserelin ($P < 0.05$, Table 1 and Figure 3C). Neither treatment altered concentrations of NA cat D. Three months' treatment with goserelin reduced serum oestrogen concentrations from 231.2 pmol $l^{-1}$ (59–569 pmol $l^{-1}$) to 81.3 pmol $l^{-1}$ (40–153 pmol $l^{-1}$, $P < 0.009$).

© 2000 Cancer Research Campaign

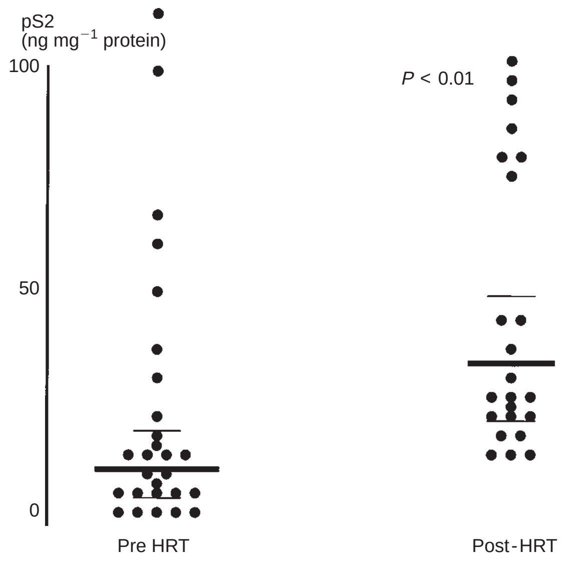

**Figure 4** The effect of hormonal replacement therapy (oestrogen alone, or oestrogen and progesterone) on the oestrogen induced protein pS2 in nipple aspirates. Sequential analysis in 26 women before and after 6 months on therapy revealed a significant rise in pS2 levels ($P \leq 0.001$)

### Effect of HRT

Secretions were obtained before, and after 6 months of HRT in 25/26 women (one individual failed to produce secretion after 6 months, HRT). Nipple aspirate concentrations of pS2 increased fourfold ($P < 0.001$, Table 2 and Figure 4) and Apo D fell significantly ($P < 0.05$) on HRT. Total protein levels in NA did not alter significantly on any treatment.

### DISCUSSION

Seventy per cent of premenopausal and 40% of post-menopausal women (Harding et al, 1996) produced NA, a yield which compares with 42% and 17% respectively reported by Wrensch et al (1990). These investigators used Sartorius cup suction, whereas we found manual compression to be equally effective. The volume of secretions varied widely: it did not increase with sequential sampling suggesting that the collection of secretions did not stimulate increased fluid production.

Proteins which are regulated by sex steroids in human breast cancer cells in vitro are detectable in most NA. The individual values vary widely reflecting differences in circulating hormone concentrations between women. When serum oestrogen concentrations were reduced by goserelin, nipple secretion concentrations of pS2 fell, whilst those of Apo D increased. Similar changes were seen with the anti-oestrogen tamoxifen and the opposite effect occurred when HRT was administered. These data suggest that pS2 and Apo D may be used to reflect the effect of oestrogens on the breast. However, changes in concentrations of cat D and CP15 were not so clear-cut.

Differences were seen in protein concentrations between normal pre- and post-menopausal women. Consistent with the post-menopausal fall in serum oestradiol concentrations, NA Cat D levels were lower and Apo D and CP15 levels were higher. The large overlap of concentrations of all measured proteins between pre- and post-menopausal women may be related to the reported high oestrogen concentrations in breast tissue and NA in post-menopausal women (Ernster et al, 1987). In women with benign disease, there was a reduction in both pS2 and Cat D after the menopause: however, Apo D and CP15 did not show the expected increase in concentrations, which may reflect the fact that breast aromatization of oestrogen continues after the menopause and causes continued suppression of Apo D and CP15 secretion although not stimulating an increase in pS2 and Cat D levels.

We have looked for biomarkers of target organ sensitivity to hormones and hypothesized that benign disorders might be related to increased sensitivity to oestrogen. Although we found higher concentrations of NA pS2 were present in premenopausal women there were no differences in NA concentrations of other proteins studied in premenopausal women. In post-menopausal women Apo D and CP15 were significantly lower in the benign group which may reflect heightened sensitivity to oestrogen suppression by low levels of oestradiol in women with benign disorders.

The best evidence for our hypothesis was found in the women with cyclical mastalgia where breast pain and tenderness can be relieved either by reducing serum oestradiol concentrations by ovarian suppression or by using anti-oestrogens suggesting that this condition is related to sensitivity of the breast to oestradiol. No consistent differences in serum oestradiol concentrations between women with and without cyclical mastalgia have been demonstrated (Ernster et al, 1987). Increased target organ sensitivity to oestradiol is supported by our data in that NA pS2 concentrations were significantly higher and Apo D concentrations were lower in women with mastalgia compared to those without (Table 1). Treatment of these women with goserelin resulted in relief of pain and a mean reduction of oestradiol of two- to eightfold. PS2 concentrations declined threefold and Apo D increased tenfold after 3 months of treatment suggesting that the synthesis of these proteins was controlled by oestrogen. This is supported by similar responses to tamoxifen (Table 1). The elevated NA levels of pS2 and suppressed Apo D and CP15 found in these women indicate that the condition is caused by increased target organ sensitivity of the breast to oestrogen and the changes in pS2, Apo D and CP15 mirrored symptomatic relief of the pain. HRT increased NA pS2 levels over a 3-month period, whereas Cat D levels were unaltered. Concentrations of NA Cat D did not decline after goserelin or tamoxifen treatment and Cat D concentrations may be influenced by factors distinct from those governing pS2 production (Cavailles et al, 1989; Rajah et al, 1996). In cases where it is unclear whether pain is originating from the breast or chest wall behind the breast, measurement of nipple aspirate pS2 levels may prove useful to aid diagnosis.

The two cyst protein markers CP15 and Apo D proved better markers of anti-oestrogen therapy as their levels rose with treatment. However, it is notable that on HRT NA pS2 concentrations rose and Apo D concentrations fell.

Although proliferating breast cancer cell lines produce pS2 and Cat D in response to oestrogen (Cavailles et al, 1989), in vitro Cat D production is also regulated by growth factor stimulation (Cavailles et al, 1989) and the failure to show changes in NA Cat D levels in women with cyclical mastalgia may reflect differences between benign and malignant epithelium or that the therapy altered growth factor production in a different direction. Breast tissue taken at comparable times during the menstrual cycle of young women shows marked differences in proliferative indices

*© 2000 Cancer Research Campaign*

(Potten et al, 1988) and there is a wide variation in the proliferative response of normal breast tissue of different women when implanted into nude mice treated with oestradiol (Potten et al, 1988). Cathepsin D (but not pS2) concentrations in NA may reflect background proliferative activity which is also influenced by growth factors secreted locally (e.g. epidermal growth factor) (Cavailles et al, 1989).

In the group of normal post-menopausal women who were commencing HRT, oestrogen administration resulted in a significant increase of NA pS2 and decrease of Apo D after 6 months treatment demonstrating that these two proteins are the optimal markers to use for studying the oestrogenicity/anti-oestrogenicity of biological compounds. NA Cat D and CP15 which did not change are not sufficiently sensitive markers of changes in serum oestradiol.

With the current search for chemopreventative agents to reduce breast cancer incidence, non-invasive markers of the anti-oestrogen and antiproliferative effect of drugs which are being tested are necessary in these short-term studies. Apo D would appear to best fit this requirement as Apo D levels rise in response to anti-oestrogen therapy and also in response to reduced cell proliferation (Simard et al, 1990). The measurement of NA Apo D after 3-month therapy with proposed anti-oestrogens (e.g. phyto-oestrogens) should inform their effect on the breast and provide evidence of their likely effectiveness over a longer time period.

This is the first time an effect of tamoxifen on the normal breast has been demonstrated, and provides a non-invasive method of assessing the effect of anti-oestrogens on the normal breast where this endocrine therapy is being prescribed for breast cancer prevention. Since only 50% of women will have their tumours prevented by anti-oestrogen therapy (Early Breast Cancer Trialists Collaborative Group, 1992) it will be important to develop a method to monitor the effects of the drug on the breast, so that non-responders can be considered for alternative or additional therapy.

We believe sequential measurement of NA levels of pS2 and Apo D will fulfil this function and may predict which women will benefit from anti-oestrogen chemoprevention therapy. The response to breast pain therapy by anti-oestrogens was predicted by a fall in pS2 and a rise in Apo D. Measurement of nipple secretion proteins is a useful pretreatment marker of women's individual response to therapy and may prove a valuable intermediate end point of effect in the ongoing chemopreventative trials of tamoxifen. It will also prove a new non-invasive method of assessing the effect of new anti-oestrogens (e.g. ICI 182 and Raloxifene) on the normal human breast.

In conclusion, we have developed a non-invasive measure of breast responsiveness to oestrogen which may prove valuable in assessing an individual woman's risk from breast cancer in relation to hormonal stimulation.

### ACKNOWLEDGEMENTS

We gratefully acknowledge the contribution of Dr DA Haagensen who supplied purified Apo D, CP15, anti-Apo D and anti-CP15; E Rogers, Research Technician, for her work in assaying CP15, C Charamboulos for his assistance with Apo D measurement and J Margison, HNC, Clinical Pharmacologist, for her guidance with Cat D and pS2 assays and the North West Regional Health Authority for financial support.

### REFERENCES

Bergkvist L, Adami HO, Persson I, Hoover R and Schairer C (1989) Risk of breast cancer after estrogen and estrogen-progestin replacement. *N Engl J Med* **321:** 293–297

Brown AMC, Jeltsch JM, Roberts M and Chambon P (1984) Activation of pS2 transcription is a primary response to oestrogen in the human breast cancer cell line MCF7. *Proc Natl Acad Sci USA* **81:** 6344–6348

Bulbrook RD, Hayward JL, Wang DY, Thomas BS, Clark GM, Allen DS and Moore JW (1986) Identification of women at high risk of breast cancer. *Breast Cancer Res Treat* **7:** 5–10

Cavailles V, Augereau P, Garcia M and Rochefort H (1988) Estrogens and growth factors induce the mRNA of the 52K-pro-cathepsin D secreted by breast cancer cells. *Nucleic Acids Res* **16:** 1903–1919

Cavailles V, Garcia M and Rochefort H (1989) Regulation of Cathepsin D and pS2 gene expression by growth factors in MCF7 human breast cancer cells. *Mol Endocrinol* **3:** 552–558

Chalbos D, Haagensen D, Parish T and Rochefort H (1987) Identification and androgen regulation of two proteins released by T47D human breast cancer cells. *Cancer Res* **47:** 2878–2792

Early Breast Cancer Trialists Collaborative Group (1992) Systemic treatment of early breast cancer by hormonal, cytotoxic or immune therapy. *Lancet* **339:** 1–15

Ernster VL, Wrensch MR, Petrakis NL, King EB, Miike R, Murai J, Goodson WH III and Siiteri PK (1987) Benign and malignant breast disease: initial study results of serum and breast fluid analyses of endogenous estrogens. *J Natl Cancer Inst* **79:** 949–960

Haagensen DE, Dilley WG, Mazoujian G and Wells SA (1990) Review of GCDFP-15. *Ann NY Acad Sci* **586:** 161–173

Harding C, Howell A and Bundred NJ (1996) Proceedings of the Benign Breast Disease Meeting, July 1995, Parthenon, London

Hunt K, Vessey M, Mcpherson K and Coleman M (1987) Long-term surveillance of mortality and cancer incidence in women receiving hormone replacement therapy. *Br J Obs Gynecol* **94:** 620–635

Key TJA and Pike MC (1988) The role of oestrogens and progestagens in the epidemiology and prevention of breast cancer. *Eur J Cancer Clin Oncol* **24:** 29–43

Lippsett MB and Bergenske DM (1960) Lack of effect of human growth hormone and ovine prolactin on cancer in Man. *Cancer Res* **20:** 1172–1178

MacMahon B, Cole P and Brown JB (1986) Urine oestrogens, frequency of ovulation and breast cancer risk: case control study in premenopausal women. *J Natl Cancer Inst* **77:** 613–616

Petrakis NL (1986) Physiologic, biochemical and cytological aspects of nipple aspirate fluid. *Breast Cancer Res Treat* **8:** 7–19

Petrakis NL, Ernster VL, Sacks ST, King EB, Schweltzer RJ, Hunt TK and King MC (1981) Epidemiology of breast fluid secretion: association with breast risk factors and cerumen type. *J Natl Cancer Inst* **67:** 277–284

Petrakis NL, Wrensch MR, Ernster VL, Miike R, Mirai J, Simberg N and Sitteri PK (1987) Influence of pregnancy and lactation on serum and breast fluid oestrogen levels: implications for breast cancer risk. *Int J Cancer* **40:** 587–591

Petrakis NL, Lowenstein JM, Wiencke JK, Lee MM, Wrensch MR, King EB, Hilton JF and Miike R (1993) Gross cystic disease fluid protein in nipple aspirates of breast fluid of Asian and non-Asian women. *Cancer Epidemiol Biomarkers Prev* **2:** 573–579

Potten CS, Watson RJ, Williams GT, Tickle S, Roberts SA, Harris M and Howell A (1988) The effect of age and menstrual cycle upon proliferative activity of the normal human breast. *Br J Cancer* **58:** 163–170

Rajah TT, Dunn T and Pento JT (1996) The influence of antioestrogens on pS2 and cathepsin D mRNA induction in MCF-7 breast cancer cells. *Anticancer Res* **16**: 837–842

Sanchez LM, Visozo F, Diez-Itza I and Lopez-Otin C (1992) Identification of the major protein components in breast secretions from women with benign and malignant breast diseases. *Cancer Res* **52**: 95–100

Simard J, Dauvois S, Haagensen D, Levesque C, Merand Y and Labrie F (1990) Regulation of progesterone-binding breast cyst protein GCDFP 24 secretion by oestrogens and androgens in human breast cancer cells: a new marker of steroid action in breast cancer. *Endocrinology* **126**: 3223–3231

Weaver I, Christine A, Springer PA and Katzenellenbogen B (1988) Regulation of pS2 gene expression by affinity labelling and reversibly binding oestrogens and antioestrogens. *Mol Endocrinol* **2**: 936–945

Wrensch MR, Petrakis NL, Gruenke LD, Ernster VL, Miike R, King EB and Hauck (1990) Factors associated with obtaining nipple aspirate fluid: analysis of 1428 women and literature review. *Breast Cancer Res Treat* **15**: 39–51

Zumoff B (1988) Hormonal profiles in women with breast cancer. *Anticancer Res* **8**: 627–636

© 2000 Cancer Research Campaign

## References

[PDF_00632] Bergkvist L., Adami H. O., Persson I., Hoover R., Schairer C. (1989). The risk of breast cancer after estrogen and estrogen-progestin replacement.. N Engl J Med.

[PDF_00635] Brown A. M., Jeltsch J. M., Roberts M., Chambon P. (1984). Activation of pS2 gene transcription is a primary response to estrogen in the human breast cancer cell line MCF-7.. Proc Natl Acad Sci U S A.

[PDF_00638] Bulbrook R. D., Hayward J. L., Wang D. Y., Thomas B. S., Clark G. M., Allen D. S., Moore J. W. (1986). Identification of women at high risk of breast cancer.. Breast Cancer Res Treat.

[PDF_00641] Cavailles V., Augereau P., Garcia M., Rochefort H. (1988). Estrogens and growth factors induce the mRNA of the 52K-pro-cathepsin-D secreted by breast cancer cells.. Nucleic Acids Res.

[PDF_00644] Cavailles V., Garcia M., Rochefort H. (1989). Regulation of cathepsin-D and pS2 gene expression by growth factors in MCF7 human breast cancer cells.. Mol Endocrinol.

[PDF_00647] Chalbos D., Haagensen D., Parish T., Rochefort H. (1987). Identification and androgen regulation of two proteins released by T47D human breast cancer cells.. Cancer Res.

[PDF_00652] Ernster V. L., Wrensch M. R., Petrakis N. L., King E. B., Miike R., Murai J., Goodson W. H., Siiteri P. K. (1987). Benign and malignant breast disease: initial study results of serum and breast fluid analyses of endogenous estrogens.. J Natl Cancer Inst.

[PDF_00656] Haagensen D. E., Dilley W. G., Mazoujian G., Wells S. A. (1990). Review of GCDFP-15. An apocrine marker protein.. Ann N Y Acad Sci.

[PDF_00660] Hunt K., Vessey M., McPherson K., Coleman M. (1987). Long-term surveillance of mortality and cancer incidence in women receiving hormone replacement therapy.. Br J Obstet Gynaecol.

[PDF_00663] Key T. J., Pike M. C. (1988). The role of oestrogens and progestagens in the epidemiology and prevention of breast cancer.. Eur J Cancer Clin Oncol.

[PDF_00666] LIPSETT M. B., BERGENSTAL D. M. (1960). Lack of effect of human growth hormone and ovine prolactin on cancer in man.. Cancer Res.

[PDF_00668] Meyer F., Brown J. B., Morrison A. S., MacMahon B. (1986). Endogenous sex hormones, prolactin, and breast cancer in premenopausal women.. J Natl Cancer Inst.

[PDF_00673] Petrakis N. L., Ernster V. L., Sacks S. T., King E. B., Schweitzer R. J., Hunt T. K., King M. C. (1981). Epidemiology of breast fluid secretion: association with breast cancer risk factors and cerumen type.. J Natl Cancer Inst.

[PDF_00679] Petrakis N. L., Lowenstein J. M., Wiencke J. K., Lee M. M., Wrensch M. R., King E. B., Hilton J. F., Miike R. (1993). Gross cystic disease fluid protein in nipple aspirates of breast fluid of Asian and non-Asian women.. Cancer Epidemiol Biomarkers Prev.

[PDF_00671] Petrakis N. L. (1986). Physiologic, biochemical, and cytologic aspects of nipple aspirate fluid.. Breast Cancer Res Treat.

[PDF_00676] Petrakis N. L., Wrensch M. R., Ernster V. L., Miike R., Murai J., Simberg N., Siiteri P. K. (1987). Influence of pregnancy and lactation on serum and breast fluid estrogen levels: implications for breast cancer risk.. Int J Cancer.

[PDF_00683] Potten C. S., Watson R. J., Williams G. T., Tickle S., Roberts S. A., Harris M., Howell A. (1988). The effect of age and menstrual cycle upon proliferative activity of the normal human breast.. Br J Cancer.

[PDF_00686] Rajah T. T., Dunn S. T., Pento J. T. (1996). The influence of antiestrogens on pS2 and cathepsin D mRNA induction in MCF-7 breast cancer cells.. Anticancer Res.

[PDF_00692] Simard J., Dauvois S., Haagensen D. E., Lévesque C., Mérand Y., Labrie F. (1990). Regulation of progesterone-binding breast cyst protein GCDFP-24 secretion by estrogens and androgens in human breast cancer cells: a new marker of steroid action in breast cancer.. Endocrinology.

[PDF_00689] Sánchez L. M., Vizoso F., Díez-Itza I., López-Otín C. (1992). Identification of the major protein components in breast secretions from women with benign and malignant breast diseases.. Cancer Res.

[PDF_00696] Weaver C. A., Springer P. A., Katzenellenbogen B. S. (1988). Regulation of pS2 gene expression by affinity labeling and reversibly binding estrogens and antiestrogens: comparison of effects on the native gene and on pS2-chloramphenicol acetyltransferase fusion genes transfected into MCF-7 human breast cancer cells.. Mol Endocrinol.

[PDF_00699] Wrensch M. R., Petrakis N. L., Gruenke L. D., Ernster V. L., Miike R., King E. B., Hauck W. W. (1990). Factors associated with obtaining nipple aspirate fluid: analysis of 1428 women and literature review.. Breast Cancer Res Treat.

[PDF_00702] Zumoff B. (1988). Hormonal profiles in women with breast cancer (review).. Anticancer Res.

